# Student and surgeon perceptions of a robotic theatre live-link within the undergraduate curriculum

**DOI:** 10.1007/s11701-026-04055-4

**Published:** 2026-10-07

**Authors:** Lewis Blenkinsop, Kenneth Campbell, Neil Harrison, Ross Dolan

**Affiliations:** 1 Dundee Institute of Healthcare Simulation, University of Dundee, Ninewells Hospital, Dundee, DD1 9SY UK; 2 Department of Colorectal Surgery, Ninewells Hospital, Dundee, UK

**Keywords:** Undergraduate medicine, Learning in the operating theatre, Robotic surgery, Live-link, Telelink

## Abstract

Explore student and surgeon perceptions of robotic theatre live-links as part of an undergraduate medical degree programme. During the surgical block, in the fourth year of a five-year medical programme at the University of Dundee, a robotic live-link session was introduced. Students undertook a pre-session case discussion and targeted anatomy revision before linking in to view the live operation. During the live-link session, the operating surgeon facilitated group discussion and answered questions via a headset. Following the session, 12 students and four participating surgeons completed a questionnaire exploring their experience of the session. The data collected was analysed descriptively to assess the acceptability of this teaching method to the staff and students as well as the students' perceived benefits of the learning environment. The session was well received, with 92% (11/12) of students describing it as relevant or very relevant to their learning. Students also reported fewer barriers to learning compared with their experiences in theatre, such as feeling unable to ask questions. Surgeons felt the quality of the teaching delivered was as good as or better than that normally delivered in theatre. They also reported that the live-link did not impact negatively on their ability to undertake the clinical case. This appears to be the first instance of a robotic theatre live-link being timetabled within an undergraduate curriculum. Student and faculty feedback is positive, and it may provide a way to introduce students to theatre without the usual barriers to learning.

**Supplementary Information:**

The online version contains supplementary material available at https://doi.org/10.1007/s11701-026-04055-4.

## Background

Robotic surgery has expanded rapidly over the last 20 years [1], but undergraduate education has lagged behind with curricula still in early development [2], despite high levels of student interest in the modality [3]. 

Traditionally, learning through experience in the operating theatre has been a fundamental part of an undergraduate surgical rotation [4]. However, providing valuable theatre experience for students presents challenges. From an administrative perspective, the challenge is to avoid overcrowding, particularly in the UK, where cohort sizes have rapidly grown over the preceding decade [5]. 

From an educationalist’s perspective, a valuable theatre experience is a hard concept to define. Sessions often lack learning objectives, and the surgeons in theatre may be unaware of the wider curriculum requirements. When learning objectives are set by surgeons, they tend to favour teaching clinical knowledge and anatomy in particular [6]. Students agree, reporting that clinical anatomy is the topic most heavily emphasised in the operating room [7]. Surgeons also highlight that prior reading of the case and its relevant anatomy is critical preparation for a theatre placement [6]. 

Students then face challenges of their own. They must overcome feeling intimidated in theatre [8] and having no clear role [9], while also being placed in an alien learning environment where learning is frequently secondary to patient care [10] and learning objectives are often not explicit [11]. Where objectives are set, they may differ from students’ own learning goals as they tend to prioritise practical skills over knowledge [6]. Robot-assisted theatre presents additional challenges. Students describe a more indirect relationship with the operating surgeon than in open or laparoscopic surgery and consider themselves peripheral to the case, finding little benefit in observing it on a screen within the theatre environment [12]. 

These challenges led to the introduction of live-links to robotic theatre within the fourth-year undergraduate curriculum. While the concept of live-link or telelink theatre is not new [13–15], we believe this is the first example of a live-link to robotic theatre being embedded in the undergraduate curriculum and not being only an optional extracurricular session. The same challenge that students describe, being peripheral to the case, is exactly what makes the modality ideal for such teaching sessions.

Here, we describe the design and delivery of this programme, including initial feedback from students and surgeons on the first five live-link sessions.

## Methods

All 4th-year MBChB students are timetabled for one live-link during their 8-week surgical block. This session was implemented in April 2026, with 4–7 students per session. The session lasts 60–90 min and is facilitated in the seminar room by a surgical teaching fellow. Students initially receive a 30-minute orientation lecture in which the case history is discussed, followed by revision of relevant key anatomy. This is similar to the methods described by Smeeton et al., [14] who found pre-session lectures were well received by students who signed up to live-link plastic surgery cases.

Preparation for the session requires initial discussion between the teaching fellow and the operating surgeon during the preceding week. This allows a presentation to be tailored to the specific case and a start time for the session to be agreed that maximises student exposure to the relevant anatomy or pathology. On the morning of surgery, the surgeon or teaching fellow facilitating the session speaks with the patient to explain and obtain consent to the live-link.

Upon starting, the live-link session follows a standardised four-part structure:

1. Using the view from the robotic console, the surgeon explains the procedure thus far and orientates the students to the anatomy. (5–10 min)
2. The surgeon continues the operation, answering questions from the student groups as they do so. This is facilitated by the Fellow in the room. (15–20 min)
3. The audio link is ended and an overhead camera is used for a discussion around theatre team roles, facilitated by the Fellow. (5–10 min)
4. A debrief is undertaken to identify gaps in the students’ understanding and to direct future learning. (10–15 min)

### Technology

The da Vinci Xi system (Intuitive Surgical, Sunnyvale, CA) is used in the theatre, and the AV system linking to the seminar room was designed and installed by Streamtec (Dundee, Scotland).

In the operating theatre, this consists of a wall-mounted camera, a headset for the surgeon, a video encoder, and a wireless receiver. In the seminar room, a second headset and wireless receiver are required, along with two video decoders and a picture-in-picture processor.

### Feedback

Students were invited to complete a questionnaire administered via the Online Surveys platform (Jisc; Joint Information Systems Committee, Bristol, UK) (Supplementary 1) consisting of 14 questions which were a mix of rank choice, Likert rating and free text response. Responses to these questions were analysed to assess students’ perceptions of the session and how it compared to traditional teaching modalities.

Consultants who took part were invited by email at the end of the block to complete a feedback questionnaire, hosted on the same system (Supplementary 2) with the same question mix. Responses were analysed to assess any challenges the surgeons experienced that may have impacted on their ability to operate safely and how these compared to students’ traditional theatre placements.

## Results

### Students

A total of five live-links took place over the data collection period. Of the five links, two were urological operations, two were gynaecological, and one was colorectal. A total of 27 students were timetabled for the sessions; however, attendance was not directly recorded.

Feedback has been collected from 12 of the students, all of whom had been to theatre at least five times and scrubbed in theatre at least once. Students described the same challenges to learning in theatre as described in the literature, such as feeling intimidated and unable to ask questions. Four students had seen robotic surgery before, and 92% (11/12) felt the session was relevant or very relevant to their learning.

When asked to rank the value to their education of different modalities of surgical teaching (Table 1), the live-link was consistently the second-choice modality. It was occasionally ranked above time in theatre.

**Table 1:** Frequency of students’ responses to the question “*rank these teaching modalities in order of value to your surgical education*”. Values are the number of students assigning each rank; 1st indicates the modality ranked most valuable

Modality	1st	2nd	3rd	4th	5th
Time in theatre	9	0	1	1	1
Live-link	1	9	2	0	0
Time using simulator	2	2	7	0	1
Lecture alone	0	1	2	6	3
Pre-recorded theatre session	0	0	0	5	7

With the live-link, students appreciated the anatomy explanation and felt more able to ask questions during the session than in the theatre. The free-text question “What did you learn during this session?” received ten responses, six of which mentioned the case anatomy as something they had learned. One response instead described the setting: “…seeing it in an environment when we can ask lots of questions is nice” (Table 2).

### Surgeon

Of the five surgeons who took part, four completed feedback. All agreed that the live-link is an effective way to teach undergraduates and were able to teach without compromising their operative focus.

All felt operative efficiency was unchanged compared with having students in theatre and agreed that the quality of teaching was as good as or better than that delivered in theatre.

Three of the four surgeons ranked the live-link as the most valuable modality for a student’s surgical education; the fourth ranked it second, behind time in theatre.

**Table 2:** Individual rankings by each of the four participating surgeons in response to the question *“rank these teaching modalities in order of value to students’ surgical education”*. 1st indicates the modality ranked most valuable

Surgeon	1st	2nd	3rd	4th	5th
S1	Live-link	Theatre	Pre-recorded	Simulator	Lecture
S2	Live-link	Pre-recorded	Lecture	Simulator	Theatre
S3	Theatre	Live-link	Pre-recorded	Lecture	Simulator
S4	Live-link	Pre-recorded	Simulator	Theatre	Lecture

## Discussion

Previous studies on live-link theatre have been limited to extracurricular sessions, resulting in a self-selecting group of learners. These studies report higher levels of student engagement [13] and student preference over traditional theatre [14, 16]. 

Our work demonstrates the feasibility of delivering this format across an entire undergraduate cohort, and its acceptability to both students and surgeons. Students particularly appreciated the pre-session lecture, which oriented them not only to the case but also to the operative approach and key anatomy, and from a student perspective this appears to be a vital component of the session. This mirrors the preparation consultant surgeons identify as most important before a theatre placement, but which students rarely manage to complete [6]. 

Moving students out of the operating theatre may also allow a session to commit to a single objective. In theatre, a student may focus on scrubbing and assisting, an objective many surgeons may not facilitate [6]. By removing the prospect of practical participation altogether, the live-link allows the session to concentrate on the anatomy and operative understanding that surgeons set as their objective and that students themselves recognise theatre teaches well [6, 7]. 

Operating surgeons felt the sessions were worthwhile and did not impede their operating. Three of the four rated the sessions more valuable to students than being in theatre physically.

Robotic cases were chosen initially because the audiovisual system was already integrated and governance in place. In practice, however, the modality appears well suited for such sessions. Students in robotic theatre are already peripheral to the case, often watching the operation on a screen, and can find it difficult to ask questions [12]. In these sessions that screen is moved to a more familiar environment and the questioning facilitated by a dedicated member of staff. Students found asking questions easier than when in theatre and commented on the environment that the session was held in. This may reflect both the more familiar physical environment and the learning environment created by the pre-link lecture, which oriented them to the case and its anatomy.

The literature describes approaches to live-link theatre ranging from dedicated technology systems [16] to a laptop webcam [15]. Systems such as the Intuitive Hub are now making the capture and streaming of robotic theatre more straightforward, and it is likely that live-link theatre will become easier to facilitate in the coming years, depending on local governance practices. There is no reason why a similar method could not be employed in laparoscopic surgery, although the benefits may be less pronounced due to inferior operator ergonomics [1], the requirement for a separate camera operator and the lack of regular microphone use by the surgeon.

The key limitations of this study are its sample size and the student self-selection inherent in any optional feedback activity, as it may be that only the most engaged students completed the feedback. With only 12 respondents out of a timetabled 27 students, and with attendance not recorded, care should be taken before generalising to other centres. Sample size is a particularly pertinent issue in consultant feedback where of the four consultants one is a co-author. In addition to these, this paper presents no direct comparison group and uses a non-validated questionnaire.

The findings reported are also of perceptions rather than measured learning and while free text responses mentioned learning the case anatomy, we feel this study sits on the first level of the Kirkpatrick model of training evaluation. A formal pre- and post-session assessment of case-specific knowledge would be the next step in this programme’s evaluation.

## Conclusion

Live-links to robotic theatre provide an opportunity to introduce students to learning in the operating theatre while mitigating some of the recognised challenges there. The modality enables larger groups of students to learn in a semi-structured environment and may help better prepare them for in-theatre experiences. Importantly, the live-link was not reported to impede the surgeon’s focus on the clinical case.

## Supplementary Information

Below is the link to the electronic supplementary material.

Supplementary Material 1

Supplementary Material 2

## Data Availability

Data presented here can be made available by contacting the corresponding author.
