# Supplementary material for: Student and surgeon perceptions of a robotic theatre live-link within the undergraduate curriculum
Source: J Robot Surg. 2026 Oct 7;20(1):1069. doi: 10.1007/s11701-026-04055-4 (PMC13645965; doi:10.1007/s11701-026-04055-4)
Supplement: Supplementary file 1 [file 11701_2026_4055_MOESM1_ESM.pdf]

Robotic live link

Thank you for your interest in the study

Invitation to take part

You are being invited to take part in a research study exploring how changes to the undergraduate surgical curriculum influence students' learning in the operating theatre. Before you decide whether to take part, it is important for you to understand why the research is being done and what it will involve.

What is the purpose of the study?
The aim of this study is to explore whether increased use of surgical simulation helps students gain more educational value from their time in the operating theatre and engage with learning at a deeper level. The findings may be used to improve the undergraduate surgical curriculum and may be published in academic journals or presented at conferences.

Why have I been invited?
You have been invited because you are a Year 4 medical student who is currently on their surgical block.

Do I have to take part?
Participation is entirely voluntary. Choosing not to take part will not affect your teaching, assessments, academic progress, or your relationship with the University. You are free to withdraw at any time without giving a reason.

What will happen if I take part?
If you agree to take part, you will be asked to complete a questionnaire detailing your experience of the robotic live link. Questions should take less than 5 minutes to complete.

Are there any risks or benefits?
There are no anticipated risks associated with taking part. While there may be no direct personal benefit, your participation will help improve the surgical placement for future students.

Confidentiality and data protection
All information collected during this study will be kept strictly confidential and handled in accordance with the UK General Data Protection Regulation (UK GDPR) and the Data Protection Act 2018

What will happen to the results?
The anonymised results may be published in academic journals or presented at conferences. No information that could identify you will be included in any reports.

Further information
If you have any questions about the study, please contact:

Lewis Blenkinsop – lblenkinsop001@dundee.ac.uk

I confirm that I have read and understood the Participant Information provided on the prior page and have had
the opportunity to ask questions.

I understand that my participation is voluntary and that I am free to withdraw at any time
without giving a reason and without any impact on my teaching or relationship with the
University.

I understand that participation or non-participation will have no effect on my academic
progress or assessments.

I understand that my responses to questions will be anonymised and that no identifiable information will be used in any reports or publications.

I understand that anonymised data may be quoted in research outputs.

I understand that all data will be stored securely in accordance with UK GDPR and
University data protection policies.

I understand that all identifiable data will be destroyed within one year of collection.

I understand that only the research team will have access to the data.

1. Based on the information above are you happy to take part in the study?
*

- ◯ Yes
- ◯ No

2. What is your identified gender?

- ◯ Male
- ◯ Female
- ◯ Other

3. What is your age?

0/32,000 characters

4. Have you seen robotic surgery before? If yes how did this experience compare?

0/32,000 characters

5. How enjoyable was the session? (1 not enjoyable 5 very enjoyable)

1	2	3	4	5

Not enjoyable — Very enjoyable

6. How relevant to your learning was this session? (1 not relevant 5 very relevant)

1	2	3	4	5

Not relevant — Very relevant

7. What did you learn during this session?

0/32,000 characters

8. Will this session change your behaviour the next time you are in theatre? How so?

0/32,000 characters

9. How were the timings of the session (time spent teaching, time spent linked, time for debrief)?

0/32,000 characters

10. What would you change for this session in the future?

0/32,000 characters

11. How many times have your previously been in any operating theatre? For how many of these were you scrubbed?

0/32,000 characters

12. When you have been in theatre previously do you face any barriers to learning?

Describe these barriers and discuss if todays session impacts them.

0/32,000 characters

13. Rank these teaching methods in order of value to your surgical education

- Lecture alone
- Live-Link session (this session)
- Pre-recorded theatre session
- Time in theatre

- Time using simulator (laparoscopic/robot etc...)

14. Any other comments?

0/32,000 characters
