# Supplementary material for: Student and surgeon perceptions of a robotic theatre live-link within the undergraduate curriculum
Source: J Robot Surg. 2026 Oct 7;20(1):1069. doi: 10.1007/s11701-026-04055-4 (PMC13645965; doi:10.1007/s11701-026-04055-4)
Supplement: Supplementary file 2 [file 11701_2026_4055_MOESM2_ESM.pdf]

Consultant Faculty Survey: Robotic Live-Link Teaching for Year 4 Medical Students

Thank you for taking part in this evaluation of the robotic surgery live-link teaching sessions for Year 4 medical students at the University of Dundee.

The aim of this short survey is to gather faculty perspectives on the educational value, operative impact, and practical delivery of the live-link teaching modality. Your responses will help refine the sessions and contribute to a planned publication describing this teaching innovation.

The survey should take approximately 5–8 minutes to complete.

A note on confidentiality: Because only five consultants have participated in delivering these sessions, complete anonymity cannot be guaranteed. Responses will be reported in aggregate, and any direct quotations used in publication will be non-attributed (i.e., not linked to your name, specialty, or any identifying feature). Where a quotation might inadvertently identify a respondent, we will either paraphrase it or seek your explicit permission before use.

You may withdraw from the survey at any point before submission by closing the browser window.

1. I confirm that:
   I have read and understood the information above
   I understand that my responses may be used in an academic publication describing this teaching modality
   I understand that quotations will be reported on a non-attributed basis as described above
   I consent to my responses being used for this purpose

   *

- ◯ I confirm
- ◯ I do not confirm (I do not consent to my data use)

2. What is your surgical speciality?

0/32,000 characters

3. Years since CCT (or equivalent):

0/32,000 characters

4. For how long have you been independently operating with the robot?

0/32,000 characters

5. Before participating in this initiative, had you delivered teaching to students or trainees via a live video link from theatre? If yes tell us about previous experiences and how this compared

0/32,000 characters

6. The live-link format is an effective way to teach undergraduate students about robotic surgery.
(1 = Strongly disagree, 2 = Disagree, 3 = Neither agree nor disagree, 4 = Agree, 5 = Strongly agree)

1	2	3	4	5

Strongly disagree — Strongly agree

7. Students appeared engaged during the session.

1	2	3	4	5

Strongly disagree — Strongly agree

8. Students asked more or better-quality questions than they typically would when physically present in theatre.

1	2	3	4	5

Strongly disagree — Strongly agree

9. The educational value of these sessions justifies the time and effort involved in delivering them.

1	2	3	4	5

Strongly disagree — Strongly agree

10. Rank these teaching methods in order of value to a student's surgical education (1 most valuable, 5 least)

- Lecture alone
- Live-Link Session (this session)
- Pre-Recorded Theatre Session
- Time in Theatre

- Time using a simulator (laparoscopic/robotic etc.)

11. I was able to teach effectively during the session without compromising my focus on the operation.
(1 = Strongly disagree, 2 = Disagree, 3 = Neither agree nor disagree, 4 = Agree, 5 = Strongly agree)

1	2	3	4	5

Strongly disagree Strongly agree

12. The live-link added significant cognitive load while I was operating.

1	2	3	4	5

Strongly disagree Strongly agree

13. The session disrupted the flow or timing of the operating list.

1	2	3	4	5

Strongly disagree Strongly agree

14. Were there any moments during the session where the teaching role conflicted with the surgical task or altered your surgical approach? If so, please describe briefly.

0/32,000 characters

15. Compared to having students in theatre how did the quality of teaching delivered compare?

1	2	3	4	5

Much worse Much better

16. Compared to having students in theatre, how did the efficiency of the theatre session feel?

1	2	3	4	5

Much worse Much better

17. Compared to having students in theatre, how did student engagement feel?

1	2	3	4	5

Much worse Much better

18. Please share any reflections on the patient consent process for live-link sessions, including any issues encountered or tips for colleagues.

0/32,000 characters

19. Was there any part of the session that you thought worked particularly well, if so why did it?

0/32,000 characters

20. Do you have any suggested improvements for the session?

0/32,000 characters

21. How happy would you be to take part in further live-link sessions?

- ◯ I would happily take part
- ◯ I would take part with some modifications (please outline above)
- ◯ Unsure
- ◯ I would not take part
